# Arteriovenous fistula in the head and neck - a systematic review and meta-analysis of clinical presentation

**DOI:** 10.1007/s00405-025-09644-x

**Published:** 2025-08-29

**Authors:** Jurriën L. A. Embrechts, Rilke J. Snoeren, Joseph C. J. Bot, Birgit Lissenberg-Witte, Johannes C. F. Ket, C. Rene Leemans, Remco de Bree

**Affiliations:** 1 Department of Otolaryngology - Head and Neck Surgery, Amsterdam UMC, Location VUmc, Amsterdam, Netherlands; 2 Department of Otolaryngology - Head and Neck Surgery, Christchurch Public Hospital, Christchurch, New Zealand; 3 Department of Pulmonology, Hagaziekenhuis, The Hague, The Netherlands; 4 Department of Radiology and Nuclear Medicine, Amsterdam UMC, Location VUmc, Amsterdam, Netherlands; 5 Medical Library, Vrije Universiteit, Amsterdam, Netherlands; 6 Amsterdam UMC, Location VUmc, Epidemiology & Data Science, Amsterdam, Netherlands; 7 Department of Head and Neck Surgical Oncology, University Medical Center Utrecht, Utrecht, Netherlands

**Keywords:** Arteriovenous fistula, Arteriovenous anomaly, Head and neck

## Abstract

**Objective:**

To identify clinical characteristics, anatomical distribution and risk factors of arteriovenous fistula in the head and neck area (hAVF).

**Methods:**

A systematic review and meta-analysis on individual participant data of available literature from inception to September 2024 on extracranial and extradural hAVF was performed.

**Results:**

The systematic search resulted in an inclusion of 869 cases with a median age of 35 years and 58.5% being male. Overall 36.5% patients with a primary hAVF and 63.5% with a secondary hAVF were observed. The most common symptom at presentation is objective sound (thrill, bruit or murmur) in 59.8% patients. The vertebral artery (32.8%), superficial temporal artery (20.4%) and brachiocephalic or subclavian artery (10.1%) were the most common affected afferent vessels in the hAVFs. The internal (31.0%) and external (5.2%) jugular and brachiocephalic or subclavian vein (9.6%) are the most common affected efferent vessels. In the secondary group 43.1% developed the hAVF after invasive treatment of which 34.0% after insertion of a central venous catheter in the internal jugular vein, 8.4% after Implantable Cardioverter Defibrillator (ICD) or pacemaker lead removal, 4.2% after a hemodialysis catheter and 7.1% after hair transplantation surgery.

**Conclusions:**

hAVFs are rare vascular malformations that can be differentiated into primary (congenital or spontaneous) and secondary (traumatic or iatrogenic) lesions. Patients often present with an objective sound (thrill, bruit or murmur) and symptoms that can be related to specific vessels. Secondary hAVF is almost twice as frequent compared to primary hAVF. There is a sex predilection of secondary hAVF towards males presenting more often with a traumatic hAVF. Iatrogenic risk factors such as intravenous catheter placement, hemodialysis catheter and pacemaker lead removal and hair transplantation surgery may contribute to the development of a secondary hAVF.

**Supplementary Information:**

The online version contains supplementary material available at 10.1007/s00405-025-09644-x.

## Introduction

Vascular anomalies consist of a broad and heterogeneous spectrum of both vascular tumors and vascular malformations. Both tumors and vascular malformations may show fast or slow intralesional blood flow. Slow-flow lesions consist of capillaries, lymphatic vessels, veins or a combination. Fast-flow lesions include all lesions with increased arterial flow to and through the lesion, usually resulting in secondary local or regional venous congestion, and are potentially more aggressive [1, 2]. Arteriovenous fistulas (AVFs) were first recognized by William Hunter in 1757 and are currently classified as aggressive high-flow type simple vascular malformations according to the ISSVA (International Society for the Study of Vascular Anomalies) classification 2018 [3, 4]. They consist of a direct connection between an artery and a vein without an intervening capillary bed and may behave similarly as arteriovenous malformations (AVMs), however AVM are more complex lesions and a nidus of dysplastic vessels may be present between feeding arteries and draining veins [7]. Symptoms resulting from AVFs in the head and neck area (hAVFs) depend on lesion location, lesion shunt velocity and may result in disfigurement, (distal) ischemia with or without functional loss, pain, pulsatile tinnitus and (lethal) cardiac decompensation in severe cases. In 1875 Nicoladoni described a decrease in heart rate after compression of the feeding artery proximal to an AVF illustrating the significant hemodynamic changes an AVF can produce. This was further elaborated by Branham, and is therefore known as the Nicoladoni-Branham sign [8, 9]. The current literature divides hAVFs into two main groups: primary and secondary. Primary hAVFs consist of congenital or spontaneous lesions. The ISSVA classification for vascular anomalies shows the similarity in contributing genes in congenital AVF and AVM [2, 4, 10]. These genetic abnormalities may contribute to the presence of congenital AVF and AVM in syndromes such as hereditary hemorrhagic teleangiectasia, capillary malformation-arteriovenous malformations (CM-AVM), Parkes-Weber syndrome and Cowden syndrome. AVF have also been reported in spinal arteriovenous metameric syndrome (SAMS) or Cobb syndrome [2, 11–14]. Secondary AVFs are frequently caused by penetrating traumatic, iatrogenic vascular injury during endovascular procedures or surgery and can be accompanied by a pseudoaneurysm [15]. An arterial pseudoaneurysm, otherwise known as a false aneurysm, is an uncommon but well-known condition that can occur at any arterial site after an arterial puncture, which results in a locally contained turbulent blood flow forming a hematoma with a neck that typically does not close spontaneously [16]. To further identify clinical characteristics, anatomical distribution and risk factors of these different types of hAVFs, we performed a systematic review and meta-analysis.

## Methods

### Study selection

This review is reported according to the Preferred Reporting Items for Systematic Reviews and Meta-Analysis (PRISMA)-statement (www.prisma-statement.org). A systematic search on the Cochrane database, PubMed, Embase.com and Clarivate Analytics/Web of Science Core Collection from inception up to 2 September 2024 (by JLAE, RS and JCFK). The following terms were used (including synonyms and closely related words) as index terms or free-text words: ‘arteriovenous fistula’ and ‘head and neck’. The full search strategy for all databases is available in the supplementary information. Duplicate articles, articles not in English, French, German Italian or Spanish were excluded. Two independent researchers screened titles and abstracts on potentially relevant studies using following inclusion criteria: recorded or deductable etiology of lesion, no presence of a nidus, not more than one fistula, extracranial and/or extradural AVFs in head and/or neck, spinal extradural AVF, cirsoid aneurysm, AVF confirmed by imaging or histology and restricted to only human subjects. In the case of disagreement, the last author of the paper was consulted. Subsequently we examined the full text for final inclusion. We excluded studies when no full text was available, where the AVF was purposely surgically constructed, when no individual patient data was available, when etiology was not described or when the anatomical location of the AVF was unclear, not extra-cranial, not extra-dural or not in the head and neck region.

### Data extraction

Individual patient data from the included papers was collected, pooled and analysed. Parameters included were patient characteristics (age, sex) and genesis of the AVF. Genesis was divided in primary or secondary origin. From the included papers we extracted other fistula characteristics: afferent arterial feeders and efferent venous drainage, presence of a pseudoaneurysm and symptoms at presentation.

### Data analysis

R version 4.5.1 (2025-06-13) was used to pool and analyze the individual patient level data. Frequency and percentage are described for categorical variables and mean values with their standard deviation for continuous variables. Primary hAVFs were compared with secondary hAVFs for gender, age at presentation, afferent arterial feeders, efferent venous drainage and symptoms. An independent two-sided t-test was used to compare normally distributed continuous variables, Mann Whitney U test for non-normally distributed continuous variables and Fisher’s exact tests for comparison of categorical variables. Statistical significance was defined at a p-value < 0.05. An analysis on all available data from included individual patient information was performed. Holm’s method, a multiple test correction method closely related to Bonferroni but less conservative, was used to correct for multiple testing [17].

## Results

**Fig. 1:**
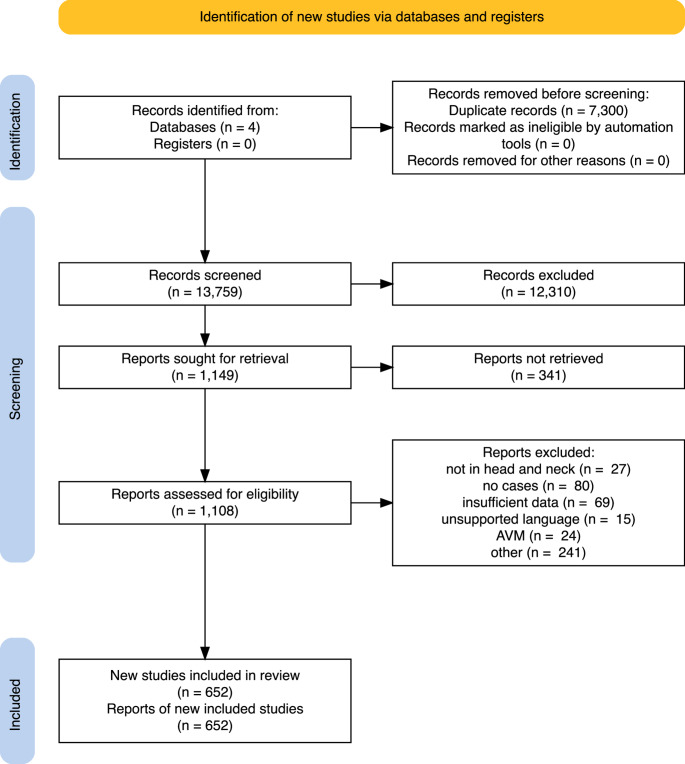
Prisma flowchart of study selection

The systematic search yielded 21,029 articles and 13,759 articles after deduplication. Selection on title and abstract eliminated 12,310 articles after consensus between the reviewers. We screened the residual 1449 articles and included 652 articles, describing 869 patients. The PRISMA-flowchart of the study selection is shown in Fig. 1.

### Patient characteristics

Two main groups are distinguished, primary and secondary hAVF with their respective subgroups. In Table 1 the patient characteristics between the two groups are compared. Median age at presentation is 35 years (interquartile range 30 years, oldest patient is a 97 year old female, the youngest a 27-week-old fetus). There was a sex predilection in the secondary group with 508 out of 869 (58.5%) being male (*p* < 0.001) due to traumatic hAVFs being more frequent in males 254/310 (82%). Patients presented with a median of 5 months after the onset of the first symptoms. We observed 317 out of 869 (36.5%) patients with a primary hAVF (congenital or spontaneous). In 94 out of 317 (29.7%) patients the lesions were congenital and the diagnosis was made at a median age of 15 years. Cirsoid aneurysms occurred significantly more often in the primary group (*p* < 0.001). A history of neurofibromatosis type 1 was significantly more frequent in the primary compared to the secondary group (*p* < 0.001), 15 out of 317 (4.7%) versus 5 out of 552 (0.9%) respectively. None of these hAVFs were diagnosed at birth.

**Table 1:** Demographics of the population. Percentages of the primary and secondary group are relative tot the total population. Percentages noted in congenital and spontaneous columns are relative to the primary group and iatrogenic and traumatic relative to the secondary group. Values followed by brackets are median value followed by their interquartile range between the brackets. NA means the value is not applicable

Characteristic	Primary		Secondary		
	Congenital *N* = 94^1^	Spontaneous *N* = 223^1^	Traumatic *N* = 314^1^	Iatrogenic *N* = 238^1^	***p***-value ^2^
Age	15 (5, 27)	42 (24, 56)	30 (23, 43)	46 (32, 61)	0.001**
Sex					< 0.001***
—	0/91 (0%)	1/218 (0.5%)	0/310 (0%)	0/237 (0%)	
Female	44/91 (48%)	111/218 (51%)	56/310 (18%)	136/237 (57%)	
Male	47/91 (52%)	106/218 (49%)	254/310 (82%)	101/237 (43%)	
Time since first symptoms (months)	1 (0, 48)	9 (2, 39)	4 (1, 36)	3 (1, 24)	0.28
Time since trauma (months)	0 (0, 0)	0 (0, 1)	4 (0, 60)	2 (0, 24)	
Reported growth	53/94 (56%)	74/223 (33%)	133/314 (42%)	85/238 (36%)	0.93
Aneurysm	15/94 (16%)	32/223 (14%)	111/314 (35%)	46/238 (19%)	< 0.001***
Cirsoid aneurysm	12/94 (13%)	38/223 (17%)	24/314 (7.6%)	5/238 (2.1%)	< 0.001***
Neurofibromatosis type 1	0/94 (0%)	15/223 (6.7%)	3/314 (1.0%)	2/238 (0.8%)	< 0.001***

^1^Median (Q1, Q3); n/N (%)

^2^Primary vs. Secondary hAVF, Fisher exact for numeric, χ² for categorical variables

A secondary hAVF (traumatic or iatrogenic) was reported in 552 out of 869 (63.5%) patients. From this group 314 (56.9%) reported a blunt or penetrating trauma in their history and 238 (43.1%) patients underwent invasive treatment. Patients that had a history of invasive treatment demonstrated in 81 out of 238 (34.0%) development of a hAVFs after insertion of a central venous catheter in the internal jugular vein and presented with a median interval of 0.9 [0.0–24.0] months after central venous cather placement. The remaining portion of these iatrogenic cases comprised 20 out of 238 (8.4%) after implantable cardioverter defibrillator (ICD) or pacemaker lead removal, in 17 out of 238 (7.1%) after hair transplantation surgery and 10 out of 238 (4.2%) after hemodialysis. A high proportion of young males was present in the traumatic group with 123 out of 314 (39.2%) being males under 30 years of age. A pseudoaneurysm was almost exclusively and significantly more present in patients with a secondary type of hAVF (*p* < 0.001).

### Clinical presentation

The clinical presentation of patients with hAVF is demonstrated in Table 2. Most patients, 680 (78.3%), presented with two or more symptoms and 24 (2.8%) were asymptomatic. The most common symptom at presentation was objective sound (thrill, bruit or murmur) in 520 (59.8%) patients. Other common symptoms were (pulsatile) tinnitus 215 (24.7%), pulsatile mass 186 (21.4%), headache 153 (17.6%) and a neurological deficit 107 (12.3%). Neurologic deficit, ocular symptoms, headache and pain were significantly more frequent in the primary group compared to the secondary group, *p* = 0.002, *p* < 0.001, *p* = 0.022 and *p* = 0.044 respectively after Bonferroni-Holm correction for multiple testing. An objective sound was more frequent in the secondary group, *p* = 0.014.

**Table 2:** Clinical presentation of primary (congenital and spontaneous) and secondary (traumatic and iatrogenic) hAVF

Characteristic	Primary^1^	Secondary^1^	*p*-value^2^	q-value^3^	Arteries	Veins
Intracranial symptoms	29 (3.4%)	50 (3.3%)	> 0.9	> 0.9	VA (64.6%), ICA (11.4%), CCA (8.9%)	IJV (36.7%), PVP (20.3%), EVP (15.2%)
Extracranial bloodloss	20 (2.3%)	49 (3.3%)	0.2	> 0.9	VA (23.2%), STA (8.7%), CCA (8.7%)	IJV (21.7%), EVP (11.6%), FV (7.2%)
Neurologic deficit	90 (11%)	93 (6.2%)	< 0.001	**0.002**	VA (61.2%), SA (8.2%), CCA (7.1%)	IJV (27.9%), EVP (17.5%), PVP (15.3%)
Visual impairment	12 (1.4%)	15 (1.0%)	0.4	> 0.9	VA (37%), OA (14.8%), OPA (14.8%)	IJV (25.9%), SOV (25.9%), IOV (14.8%)
Ocular symptoms	38 (4.4%)	23 (1.5%)	< 0.001	**< 0.001**	OA (23%), STA (19.7%), OPA (19.7%)	SOV (45.9%), IJV (21.3%), FV (8.2%)
Cardiac symptoms	27 (3.2%)	50 (3.3%)	0.8	> 0.9	SA (24.7%), CCA (15.6%), ECA (13%)	BCV (23.4%), IJV (20.8%), SV (14.3%)
Subjective sound	67 (7.8%)	150 (10%)	0.078	0.6	VA (37.8%), STA (30%), OA (12.4%)	IJV (25.8%), STV (16.6%), VV (10.6%)
Objective sound	230 (27%)	499 (33%)	0.001	**0.014**	VA (31.3%), STA (19.5%), CCA (13%)	IJV (31.1%), STV (11.8%), VV (10%)
Visible change	53 (6.2%)	96 (6.4%)	0.8	> 0.9	STA (35.6%), OA (13.4%), ECA (12.1%)	IJV (26.2%), STV (16.8%), FV (12.8%)
Headache	73 (8.5%)	79 (5.3%)	0.002	**0.022**	STA (38.8%), VA (33.6%), OA (13.2%)	IJV (24.3%), STV (16.4%), VV (8.6%)
Pain	53 (6.2%)	55 (3.7%)	0.005	**0.044**	VA (44.4%), STA (13%), OA (12%)	IJV (14.8%), EVP (13%), STV (8.3%)
Dyspnea	14 (1.6%)	42 (2.8%)	0.074	0.6	CCA (28.6%), SA (16.1%), BCA (14.3%)	IJV (42.9%), BCV (17.9%), SV (10.7%)
Pulsatile mass	76 (8.9%)	111 (7.4%)	0.2	> 0.9	STA (44.4%), OA (14.4%), VA (10.2%)	IJV (24.1%), STV (15%), FV (10.7%)
Other	73 (8.5%)	185 (12%)	0.004	**0.044**	VA (29.5%), STA (16.7%), CCA (15.9%)	IJV (31%), SV (9.3%), STV (8.9%)

^1^n (%)

^2^Pearson’s Chi-squared test

^3^Holm correction for multiple testing

### Anatomical distribution

In the primary hAVFs group the vertebral artery and branches (*n* = 133, 31.5%), superficial temporal artery (*n* = 81, 19.2%), maxillary artery and branches (*n* = 65, 15.4%), occipital artery (*n* = 50, 11.8%), vertebral vein with distal branches (*n* = 101, 24.5%) and internal jugular vein (*n* = 88, 21.3%) were the most affected vessels. In the secondary group the most common affected arteries were the vertebral artery and distant branches (*n* = 175, 26.4%), superficial temporal artery (*n* = 106, 16.0%), common carotid artery (*n* = 80, 12.1%) and the brachiocephalic artery (*n* = 76, 11.5%). The most frequent involved veins in the secondary group were the internal jugular vein (*n* = 191, 29.8%), vertebral vein and branches (*n* = 138, 21.5%) and the brachiocephalic vein (*n* = 71, 11.1%).

**Fig. 2:**
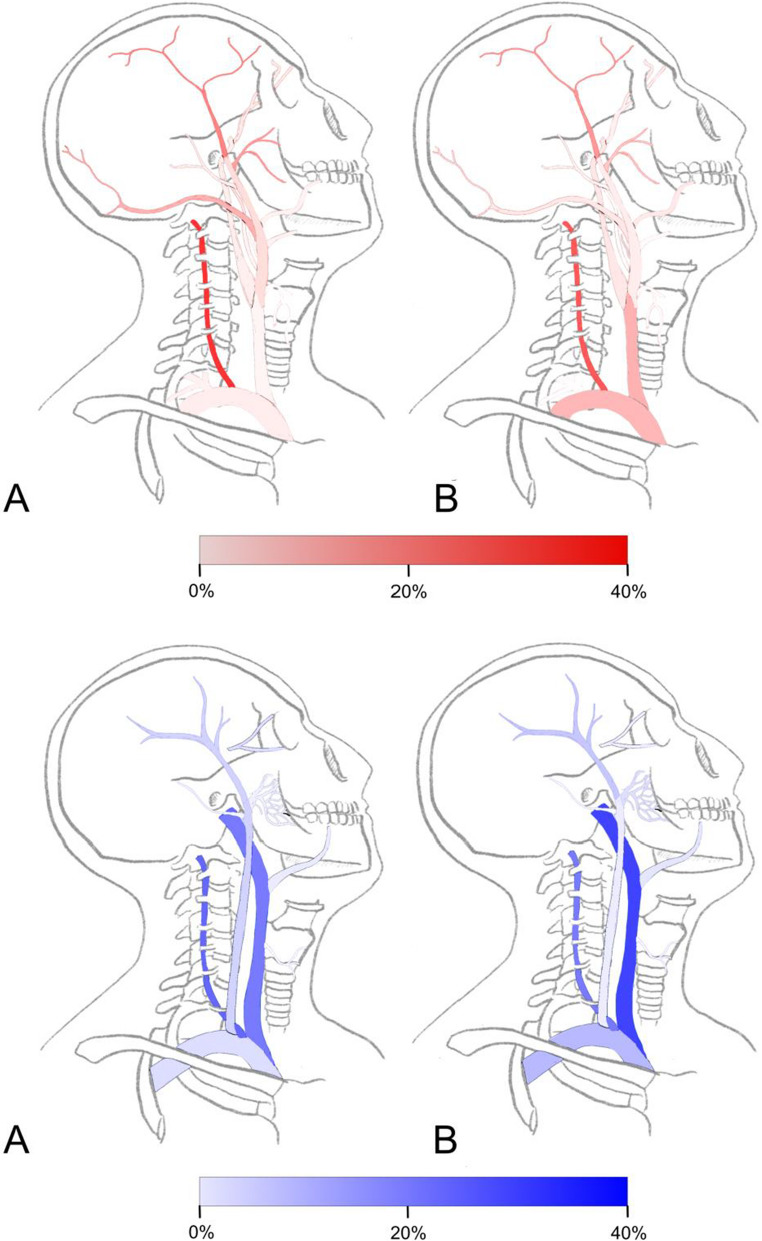
Proportions of affected arteries by locations Primary group is shown above A and secondary group above B. Vessels colored in red show the arteries and vessels colored in blue show the veins affected

The most frequently involved vessels in secondary hAVF after intravenous catheter placement were the vertebral artery in 33 out of 80 (41.2%), the common carotid artery in 22 out of 80 (27.5%) and the internal jugular vein in 41 out of 172 (23.8%) cases. After hemodialysis catheter placement the most commonly affected vessels were the carotid artery in 7 out of 80 (8.8%) and internal jugular vein in 10 out of 172 (5.8%) cases. After pacemaker lead removal we observed an involvement of the brachiocephalic and subclavian artery in 9 out of 76 (11.8%), the carotid artery in 2 out of 80 (2.5%) and the brachiocephalic or subclavian vein in 13 out of 70 (18.6%) cases. The common carotid and brachiocephalic artery as well as the brachiocephalic and internal jugular vein were significantly more affected in the secondary group compared to the primary group. Whereas the occipital and maxillary artery as well as the external jugular vein where more commonly affected in the primary group compared to the secondary group. Figure 2 provides a graphical overview of the anatomical distribution of the hAVFs and Table 3 in the supplementary information presents the proportion by location of the affected vessels and the difference between the primary and secondary group in more detail.

## Discussion

This review demonstrates a wide variety of clinical presentation in patients with hAVF varying from asymptomatic patients to life-threatening symptomatology. From an etiological perspective we can distinguish two main groups: primary (congenital or spontaneous) and secondary (traumatic or iatrogenic) AVFs.

Secondary AVFs are frequently caused by penetrating traumatic, iatrogenic vascular injury during endovascular procedures or surgery and can be accompanied by a pseudoaneurysm [15]. In this study secondary hAVFs occured almost twice as frequent compared to primary hAVFs, 552 versus 317 respectively.

A co-existent pseudoaneurysm was described in 204 out of 869 (23.5%) patients and most frequent with a traumatic secondary hAVF, additionally they can result from connective tissue disease and may occur spontaneously [18]. After penetrating trauma, pseudoaneurysm without formation of AVF is often reported (21.6–66.7%) in contrast to AVF alone (19.4%) [15, 19]. 

In this study 314 out of 869 (36.1%) of hAVF patients presented with a history of trauma that could be a potential causal mechanism for the AVF. Ito et al. showed spontaneous neo-vascularization, arteriovenous fistula formation and development of an arteriovenous shunt after patching an arterial graft into a vein [20]. A congenital or spontaneous lesion was present in 317 out of 869 (36.5%) of patients. This data is consistent with the assumption that there are at least 2 types of pathophysiological mechanisms to develop an hAVF in contrast with an AVM, which is considered to be always congenital. We observed a high rate of secondary hAVF related to iatrogenic trauma due to central venous catheter or pacemaker leads placement in these vessels as cause: In this group 41 out of 172 (23.8%) were located in the internal jugular vein, 30 out of 70 (42.9%) in brachiocephalic or subclavian veins and 24 out of 80 (30.0%) in the common carotid artery, directly related to the location of these procedures. Cronin et al. reported a complicated lead extraction with formation of an AVF in 8 of 2471 patients (0.3%). These patients also had a longer lead implant duration [21]. Pseudoaneursysms can also develop after intravenous catherization without arteriovenous fistula [22]. We found that in 17 out of 20 (85.0%) NF1 patients with an AVF this fistula involved the vertebral artery. Cervical fistulae have a unique predilection to develop in patients with NF1. This finding was already noted in 1997 by Koenigsberg et al. [23].

In this study we observed a wide variety of symptoms in patients with a hAVF. In 345 out of 869 (39.7%) of patients, no symptoms were reported and in 24 out of 869 (2.8%) of patients signs of lesional growth were reported. The latter is in contrast with AVM where all lesions ultimately show progression [24]. Asymptomatic AVFs were also often reported after penetrating vertebral artery injuries [19]. Patients may experience acute symptoms with acute loss of consciousness, neurological deficit or dyspnea notably in cases of hemorrhagic events with formation of hematoma [25, 26]. We found that certain symptoms were more common in specific locations. Knowledge of these clinical characteristics may enhance early detection of these hAVFs. Within our analysis we included extracranial and extradural hAVFs, however symptoms of AVFs located in adjacent anatomical locations can be similar e.g. AVF between the ophthalmic artery and supra-orbital vein can mimic a intra-orbital AVF with drainage to the cavernous sinus [27, 28]. In a large number of cases the feeding arteries were described more accurately compared to the draining veins complicating the definition of the exact location of the AVF. We noted a high prevalence of AVF in the vertebral artery and vein in both groups. Unfortunately, the exact location of the AVF in the long segmented course of the vertebral artery or vein was often not described. One could argue that occurrence of the AVF is merely dictated by the surface of the vessel where a greater surface presents with a higher occurrence of AVF. Awareness of patient history, different symptom patterns and risk factors could facilitate early identification and avoid doctor delay in these rare hAVFs.

### Study limitations

In this article we systematically reviewed current literature on hAVF, which is limited to case reports and case series. The retrospective and non-randomized nature of the pooled data could therefore be subject to publication bias, selection bias and may lead to an underestimation of prevalence.

## Conclusion

Head and neck arteriovenous fistulas are rare vascular malformations. They can be differentiated into primary (congenital or spontaneous) and secondary (traumatic or iatrogenic) lesions. Secondary lesions are almost twice as common as primary lesions and more common in males mostly in traumatic hAVF. Symptoms may exist multiple years before patients seek medical attention. The most common symptom at presentation is objective sound (thrill, bruit or murmur), neurologic deficit, a pulsatile mass, headache and (pulsatile) tinnitus. The vertebral, superficial temporal and occipital artery were the most commonly affected afferent vessels in the hAVFs. The internal jugular, vertebral and superficial temporal vein are the most common affected efferent vessels. Important iatrogenic risk factors for secondary AVF are intravenous catheter placement, hemodialysis, pacemaker lead removal and hair transplantation surgery.

## Supplementary Information

Below is the link to the electronic supplementary material.

Supplementary file1

## Artery

BCA: Brachiocephalic artery
CCA: Common carotid artery
ECA: External carotid artery
ICA: Internal carotid artery
OA: Occipital artery
OPA: Ophthalmic artery
SA: Subclavian artery
STA: Superficial temporal artery
VA: Vertebral artery

## Vein

BCV: Brachiocephalic vein
EJV: External jugular vein
EVP: Epidural venous plexus
FV: Facial vein
IJV: Internal jugular vein
IOV: Inferior orbital vein
OV: Ophthalmic vein
PVP: Paravertebral venous plexus
SOV: Superior ophthalmic vein
STV: Superficial temporal vein
VV: Vertebral vein
SV: Subclavian vein
