# Supplementary material for: Arteriovenous fistula in the head and neck - a systematic review and meta-analysis of clinical presentation
Source: Eur Arch Otorhinolaryngol. 2025 Aug 29;283(2):661–9. doi: 10.1007/s00405-025-09644-x (PMC12987781; doi:10.1007/s00405-025-09644-x)
Supplement: Supplementary file 1 — Supplementary file1 [file 405_2025_9644_MOESM1_ESM.docx]

# Supplementary information

| **Vessel** | **Primary**^1^ | **Secondary**^1^ | **p-value**^2^ | **q-value**^3^ |
| --- | --- | --- | --- | --- |
| Arteries |  |  |  |  |
| Common carotid | 6 (1.4%) | 80 (12%) | <0.001 | **<0.001** |
| Internal Carotid | 11 (2.6%) | 24 (3.6%) | 0.4 | >0.9 |
| ↳Ophthalmic & br. | 13 (3.1%) | 15 (2.3%) | 0.4 | >0.9 |
| External Carotid | 24 (5.7%) | 19 (2.9%) | 0.020 | 0.2 |
| ↳Thyroid sup | 0 (0%) | 6 (0.9%) | 0.087 | 0.7 |
| ↳Ascending pharyngeal | 6 (1.4%) | 1 (0.2%) | 0.016 | 0.2 |
| ↳Lingual & br. | 8 (1.9%) | 6 (0.9%) | 0.2 | >0.9 |
| ↳Facial | 4 (0.9%) | 21 (3.2%) | 0.017 | 0.2 |
| ↳Occipital | 50 (12%) | 39 (5.9%) | <0.001 | **0.006** |
| ↳Posterior Auricular | 8 (1.9%) | 7 (1.1%) | 0.2 | >0.9 |
| ↳Superficial temporal | 81 (19%) | 106 (16%) | 0.2 | >0.9 |
| ↳Maxillary and br. | 65 (15%) | 55 (8.3%) | <0.001 | **0.004** |
| ↳Brachiocephalic / Subclavian | 12 (2.8%) | 76 (11%) | <0.001 | **<0.001** |
| ↳Thyrocervical trunk & br. | 8 (1.9%) | 15 (2.3%) | 0.7 | >0.9 |
| ↳Costocervical trunk & br. | 2 (0.5%) | 1 (0.2%) | 0.6 | >0.9 |
| Vertebral & br. | 133 (32%) | 175 (26%) | 0.070 | 0.6 |
| Veins |  |  |  |  |
| Brachiocephalic / Subclavian | 21 (5.1%) | 71 (11%) | <0.001 | **0.008** |
| ↳External jugular | 28 (6.8%) | 18 (2.8%) | 0.002 | **0.021** |
| ↳Retromandibular | 0 (0%) | 3 (0.5%) | 0.3 | >0.9 |
| ↳Superficial temporal | 28 (6.8%) | 56 (8.7%) | 0.3 | >0.9 |
| ↳Pterygoid plexus | 10 (2.4%) | 7 (1.1%) | 0.094 | 0.6 |
| ↳Posterior auricular | 6 (1.5%) | 2 (0.3%) | 0.063 | 0.5 |
| ↳Internal jugular | 88 (21%) | 191 (30%) | 0.002 | **0.021** |
| ↳Superior thyroid | 0 (0%) | 4 (0.6%) | 0.2 | 0.8 |
| ↳Facial | 12 (2.9%) | 24 (3.7%) | 0.5 | >0.9 |
| ↳Ophthalmic veins | 19 (4.6%) | 16 (2.5%) | 0.063 | 0.5 |
| ↳Vertebral & br. | 101 (24%) | 138 (22%) | 0.3 | >0.9 |
| ^1^n (%) | | | | |
| ^2^Pearson's Chi-squared test; Fisher's exact test | | | | |
| ^3^Holm correction for multiple testing | | | | |

Table 3: Anatomical distribution of AVF in the head and neck. The arrow (↳) indicates that the vessel is a distal branch of the previous mentioned vessel without the arrow. ‘& br.’ indicates that distal branches of the vessel are included.
